# Green Chemistry: Terminology and Principles

**DOI:** 10.1289/ehp.0900835

**Published:** 2009-09

**Authors:** Karen Peabody O’Brien, John Peterson Myers, John Warner

**Affiliations:** Advancing Green Chemistry, Charlottesville, Virginia, E-mail: kpeaobrien@embarqmail.com; Environmental Health Sciences, Charlottesville, Virginia; Warner Babcock Institute for Green Chemistry, Wilmington, Massachusetts

We are grateful to *Environmental Health Perspectives* for implicitly embracing green chemistry as a field with profound connections to the environmental health sciences. We also commend the efforts of Wilson and Schwarzman (2009) to create greater transparency and accountability around chemicals of concern. We take issue, however, with their approach to key scientific concepts and terminology—specifically their effort to change the definition of “green chemistry.” Precision in terminology is paramount for science to function; all parties to a scientific discussion must share the same set of definitions for knowledge to advance effectively.

In their review, Wilson and Schwarzman (2009) ignored the original and current definition of green chemistry, which for almost two decades has been recognized as a scientific discipline within the field of chemistry.

Defined in the early 1990s by the U.S. Environmental Protection Agency (2009) as “the design of chemical products and processes that reduce or eliminate the use or generation of hazardous substances,” green chemistry is now guided by a set of 12 principles (Anastas and Warner 1998) that are used in both research and teaching in chemis try laboratories around the world.

Based on these principles, dozens of universities around the world teach green chemistry as a science. Seven graduate programs offer degrees in green chemistry. Two established peer-reviewed scientific journals focus specifically on research in green chemistry. The impact factor of the journal *Green Chemistry* (published by the Royal Society of Chemistry) has increased from 2.5 to almost 5 over the past 5 years. More than 1,500 articles on green chemistry have been published in the scientific literature over the past 15 years.

Rather than embracing green chemistry’s widely used scientific definition, Wilson and Schwarzman (2009) instead conflate science and policy:

The laws governing the chemical enterprise help define the incentives and disincentives that guide economic behavior in the market …. We use the term green chemistry in this context: as an analytical framework that encompasses both the science of safer chemistry and the laws and policies that will motivate its development and adoption by society.

This conflation brings with it two risks. First, it undermines clarity in scientific communication, something that is especially important as the fields of environmental health and green chemistry attempt to establish cross-disciplinary collaboration. Such collaborations are likely to prove vital for both fields. Second, it saddles the intellectual and scientific enterprise of green chemistry with policy and, potentially, political baggage, as considerations of chemical policies unfold in the political arena.

We are most certainly not arguing that the science of green chemistry should not inform chemical policies. Science and policy will be more effective, however, if political actors do not muddy accepted scientific terminology in service of a political/policy agenda, no matter how noble.
