# Errata

**Published:** 2009-10

In the letter by O’Brien et al. [Environ Health Perspect 117:A385–386 (2009)], the competing financial interest declaration was incorrect. The correct declaration is as follows:

Karen Peabody O’Brien is executive director of Advancing Green Chemistry, a not-for-profit organization that receives support from several private foundations (listed online at http://www.AdvancingGreenChemistry.org/AdvancingGreenChemistry/About_Us.html) to support efforts to build the field of green chemistry. J.P. Myers is founder, chief executive officer, and chief scientist for Environmental Health Sciences (EHS), a not-for-profit organization that receives support from several private foundations (listed online at http://www.environmentalhealthnews.org/about.html) to support EHS’s mission to advance public understanding of environmental health sciences. John Warner is president of Warner Babcock Institute for Green Chemistry, a private company that applies the principles of green chemis try in the synthesis of new materials and the redesign of chemical processes.

